# Reviewing the current state of virtual reality integration in medical education – a scoping review protocol

**DOI:** 10.1186/s13643-023-02266-6

**Published:** 2023-06-20

**Authors:** Marvin Mergen, Marcel Meyerheim, Norbert Graf

**Affiliations:** Department of Pediatric Oncology and Hematology, Faculty of Medicine, Saarland University, Building 9, Kirrberger Straße 100, 66421 Homburg, Saarland Germany

**Keywords:** Digitalization, Medical education, Medical school, Medical training, Virtual reality

## Abstract

**Background:**

Due to an increasing focus of medical curricula on clinical decision-making skills, new learning tools are constantly developed. Virtual reality (VR) is one of the emerging technologies with the potential to improve health professionals’ education. Highly realistic learning experiences with repeatable training scenarios can be created within a protected environment that is independent from real patients' presence. Our project “medical tr.AI.ning” is following this approach aiming to simulate immersive virtual first-person scenarios with intelligent, interactable virtual patients. So far, VR has been mainly used in surgical training, but there is evidence for effectiveness in training different procedural skills, such as cardiopulmonary resuscitation, knowledge acquisition, and improvement of reasoning and creativity, while still being cost-effective.

The objective of this scoping review is to explore the usage and identify key areas of VR applications in the field of medical education. Furthermore, the corresponding requirements, evaluation methods and outcomes, advantages, and disadvantages will be covered.

**Methods:**

This scoping review protocol implements the updated JBI Scoping Review Methodology. In March 2022, a preliminary literature research in PubMed was performed by two independent reviewers to refine search terms and strategy as well as inclusion criteria of the protocol, accounting for actuality and scientific relevance. The final search will be conducted using PubMed, ScienceDirect, Cochrane Library, Web of Science Core Collection, and JBI Evidence Synthesis. Search, study screening, and data extraction will be done in parallel and independently by two reviewers. Discrepancies will be handled by consensus or consulting a third review author.

**Discussion:**

With this scoping review, we anticipate collating the range of application of VR in medical education while using a transparent and reproducible search strategy. This may contribute to the design and development of novel educational VR platforms and their integration into medical curricula while pointing out previous omissions and pitfalls.

**Supplementary Information:**

The online version contains supplementary material available at 10.1186/s13643-023-02266-6.

## Background

Medical education is transforming by successively focussing more on digital learning methods. Adaption and inclusion of new technologies are supported by rapid advances in computer science which enable virtual reality (VR) to cover an increasing number of use-cases for educating medical students, doctors, and nurses.

So far, in the medical context VR has successfully but mainly been used in robotic surgery training [1]. As shown by Izard et al. (2018), virtual reality tools can be used to acquire detailed knowledge about surgical procedures. This technology enables users to repeat every procedural step as many times as required regarding the individual learning progress. An approach which would otherwise not be feasible in real-world conditions [2].

A study of Guitiérrez et al. (2007) already suggests a high benefit in acquisition of knowledge through immersive VR training compared to screen-based systems by examining first-year medical students at The University of New Mexico School of Medicine. One group experienced a fully immersive VR environment using a head-mounted display (HMD), while another group tested a partially immersive VR environment on a computer screen. The results showed benefits for both groups with significantly higher knowledge gain in the fully immersive VR group [3].

Especially in life-threatening situations, repetitive training cannot be carried out without risking the patient’s life. Learning in these stressful moments is very challenging. Creutzfeldt et al. (2016) addresses these scenarios by proving that virtual training in serious games is effective for learning cardiopulmonary resuscitation [4].

Not only during pandemic situations as with SARS-CoV-2, communication skills, e.g., talking to patients who refuse vaccination, are of great importance for healthcare providers. As Real et al. (2017) showed, these skills can successfully be trained with VR simulations closing a substantial deficit in medical schools [5]. The possibility of repetitive communication training including emotional management, as well as critical thinking and clinical decision-making will obviously improve by interactive case scenarios within a secure virtual environment. This was shown in an article by Burke et al. (2016), where virtual interactive cases were integrated into a nursing educational program. Logical reasoning and creativity have been leveraged by active engagement through virtual patient interactions [6].

VR learning platforms are used by the University of Northampton and the University of Oxford for training nurses, medical students, and doctors. The feedback of participants in these universities is very positive, like the one from the Nursing Faculty of the University of Northampton: “Technological developments are allowing us to do this in a safe and supportive learning environment, focusing on immediate feedback and the opportunity to repeat the scenarios and improve over time.” The faculty of the University of Oxford stated: “Embedding VR simulation into what we do has enabled us to give a far greater number of learners access to simulation in a shorter space of time, and lets them do it as often as they like to transfer their knowledge to practice.” [7].

The practical benefit of VR has been leveraged during the SARS-CoV-2-pandemic. VR simulations are a helpful tool that can offer valuable training of, e.g., hand disinfection, nasopharyngeal swab-taking, and donning/doffing of personal protective equipment-skills that are of great importance when it comes to preventive behavior in terms of spreading SARS-CoV-2. Birrenbach et al. (2021) successfully used VR training for COVID-19-related skills, supporting evidence that “VR is a useful tool for acquiring simple and complex clinical skills” [8].

To enhance acceptance of VR technology among students, Walter et al. (2021) suggest integration of VR simulation into the curriculum of medical schools [9]. In a study of De Ponti et al. (2020), overall students’ perception of virtual training during a time when hospital access for them could not be guaranteed was very positive. Virtual simulations can secure proper medical education even if practicing in a live hospital environment is not possible [10].

Using virtual reality for educational purposes requires an expensive hardware infrastructure. But even counting in these expenses, training with VR simulations is cost-effective in the long term compared to manikin- or actor-based trainings [11].

By conducting this scoping review, we want to gain an overview of current VR usage in medical education and define requirements as well as advantages and disadvantages of integrating this technology for training health professionals.

Due to outstanding technical advances and boosted by the pandemic, research in digitalization and particularly VR has grown rapidly during the last decade, providing a reliable foundation for this review.

According to literature, the first decade of the twenty-first century was known as the “VR winter” with little public interest in this new technology. Nevertheless, there was ongoing but limited research, mostly in corporate, academic, and military research laboratories around the world. Expenses for hardware (> 35,000$ for an HMD +  > 30,000$ for tracking) and fragile infrastructure further restricted widespread usage. Around 2011, interest in consumer-grade VR renewed, mostly for entertainment. Strong technological efforts from big-name companies such as Valve, NVIDIA (and then start-up Oculus) helped to transition the HMD-based VR technology from specialized lab instruments available only to the technical elite to a mainstream mode of content consumption available to any consumer [12].

This work is conducted within the scope of the current project “medical tr.AI.ning” [13], which will be an AI-based immersive virtual reality learning platform simulating realistic clinical scenarios with the help of intelligent, interactable virtual patients, in order to enable medical students practicing clinical decision-making. This platform will be able to solve many practical and ethical problems that current medical education faces and will help to better prepare medical students for future clinical practice. With the inclusion of a dedicated authoring tool, it will be possible to compose and individualize a large variety of unique medical training scenarios including various types of diseases. Not only symptoms, but also physiological parameters, diagnostic outcomes, subliminary diagnostic data, and even the context where a consultation happens, can be varied within scenarios ranging from private practice to virtual hospitals. This will provide a new form of situated learning addressing clinical skills and their management.

During the preliminary search in PubMed, one scoping review protocol [14] and its corresponding scoping review [15] addressing a similar topic were identified. Jiang et al. investigated the usage of VR in undergraduate or preregistration medical students’ education. Their review focuses on how this technology has been applied, which VR tools were used and the provided features.

Our scoping review will go beyond the above review by answering additional questions covering how VR has been integrated in medical education and training not only for undergraduate medical students, but also further health care professionals such as physicians and nurses. In addition, reported requirements, evaluation methods, and their outcomes are pointed out as well as advantages and disadvantages of VR technologies in medical education.

In addition, it must be noted that this work is part of a major project that itself will contribute eminently to shaping the world of virtual reality in medical education, since we aim to develop a VR training platform called “medical tr.AI.ning” that will contain the highest standards of immersion and interactivity by enabling students to practice clinical reasoning with interactive, virtual patients in an authentically simulated environment.

The objective of this scoping review is to investigate use-cases of VR in medical education and to assess requirements, evaluation, and advantages and disadvantages of this technology to establish a baseline on which further VR projects can be developed, evaluated, and integrated into medical curricula.

### Review questions

Applying the PCC concept our review deals with the following aspects:

#### Participants

We investigate reviews that cover studies on VR usage in education of medical and nursing students, registered nurses, and qualified physicians.

#### Concept

We examine VR technology as a tool for health professional education while addressing research questions considering its global spread, targeted end users, reported requirements, evaluation methodology, and outcomes.

#### Context

In this scoping review, we solely focus on publications that address medical education. Since the cost of VR hardware is still high, geographical differences could play a role considering their mean income. Moreover, findings can support future endeavors in the development and integration of VR in medical education.

With this review we want to answer the following questions:

1. In which subject/curricula is VR used in medical education?
2. How often is it used for medical/nursing students, for qualified physicians, for registered nurses?
3. How many reviews are from Germany, Europe, worldwide in general published in English or German language?
4. Which technical and didactic requirements are reported for using VR in medical education?
5. Is VR evaluated in medical education? If yes, how is it evaluated and which outcomes are reported?
6. Which advantages of VR in medical education are reported?
7. Which disadvantages of VR in medical education are reported?

## Methods

The proposed scoping review will be conducted in accordance with the updated JBI methodology for scoping reviews [16]. The respective protocol for this review was developed considering the PRISMA-P 2015 checklist [doi: 10.1186/2046-4053-4-1].  

### Inclusion criteria

This scoping review will include studies which cover the application of VR in education of medical and nursing students, doctors, and nurses. Resulting from the preliminary search in PubMed, the literature search is narrowed down to only reviews on articles about corresponding studies, because they summarize the knowledge gain, developments, and directions within this research field most comprehensively and accurately.

Only publications in English and German as languages of the authors were considered.

Respecting the time needed to conduct studies and release respective publications and reviews, 2012 seems to be the earliest year with reasonable references after public interest in VR renewed in 2011.

Reviews with no focus on educational purposes, e.g., the application of VR in actual treatment and studies that focus on augmented/extended/or mixed reality are excluded.

Primarily, we want to address requirements, evaluation, advantages, and disadvantages of VR technology in medical education. Nevertheless, the overarching concept of this scoping review is to find out, how and in which medical curricula VR has been already used.

### Search strategy

The search strategy will aim to locate published studies. A three-step search strategy will be utilized in this review. First an initial individual limited search of PubMed was undertaken independently by two authors to identify articles on the topic. By comparing search terms, analyzing the text words contained in the titles and abstracts of relevant articles, and the index terms used to describe the articles, a full search strategy for PubMed, ScienceDirect, Cochrane Library, Web of Science Core Collection, and JBI Evidence Synthesis was developed (Table 1) and reviewed by a third review author. The search strategy, including all identified keywords and index terms, will be adapted for each included database and/or information source and performed by two independent reviewers. Studies published since 2012 and written in English or German will be included. The reference lists of all included sources of evidence will be screened for additional studies.

**Table 1:** Full search strategy in PubMed, conducted in March 2022

#1	"Virtual Reality"[Mesh] OR “virtual realit*”[tw] OR VR[tw]	19,800 results
#2	"Students, Medical"[Mesh] OR “Education, Medical"[Mesh] OR “medical educat*”[tw] OR “medical teach*”[tw] OR “medical train*”[tw] OR “health professional educat*”[tw] OR “health professional teach*”[tw] OR “health professional train*”[tw] OR “medical school” [tw] OR “nursing train*”[tw] OR “nursing teach*”[tw] OR “nursing educat*” [tw] OR “physician train*”[tw] OR “physician teach*” [tw] OR “physician educat*”[tw] OR “doctor train*”[tw] OR “doctor teach*”[tw] OR “doctor educat*” [tw] OR “health care professional train*” [tw] OR “health care professional educat*”[tw]	314,100 results
#3	#1 AND #2	1,734 results
#4	#1 AND #2 Filters: Review	246 results
#5	#1 AND #2 Filters: Review, English	227 results
#6	#1 AND #2 Filters: Review, English, German	234 results
#7	#1 AND #2 Filters: Review, English, German, from 2012—2022	169 results

### Study/source of evidence selection

Following the search, all identified citations will be uploaded into Mendeley Desktop (version 1.19.8, 2020) and duplicates will be removed. After a pilot test, titles and abstracts will then be screened by two independent reviewers for assessment against the inclusion criteria for the review. Potentially relevant sources will be retrieved in full, and their citation details imported into the JBI System for the Unified Management, Assessment and Review of Information (JBI SUMARI) [17]. The full text of selected citations will be assessed in detail against the inclusion criteria by two independent reviewers. Reasons for exclusion of sources of evidence at full text that do not meet the inclusion criteria will be documented. Any disagreements that arise between the reviewers at each stage of the selection process will be resolved through discussion or with an additional reviewer. The results of the search and the study inclusion process will be reported in full in the final scoping review and presented according to the Preferred Reporting Items for Systematic Reviews and Meta-analyses extension for scoping review (PRISMA-ScR) checklist, including the PRISMA2020-flow diagram [18].

### Data extraction

Data will be extracted from citations included in the scoping review by two independent reviewers using the data extraction tool of JBI SUMARI. The data extracted will include specific details about the subject or curriculum in which VR was used, which requirements had to be met, if evaluation was performed and how, its outcomes, and the described advantages and disadvantages of VR in medical education. The data extraction form will be piloted and reviewed by a third author.

A draft extraction form is provided (Table 2). The draft data extraction tool will be modified and revised as necessary during the process of extracting data from each included evidence source. Modifications will be detailed in the resulting scoping review. Any disagreements that arise between the reviewers will be resolved through discussion or with an additional reviewer. If appropriate, authors will be contacted to request missing or additional data, where required.

**Table 2:** Data extraction form

Category	Type of data
Bibliographic information	1. Author
	2. Year of publication
	3. Country of origin
	4. Objective of the study
Research questions	1. Subject/curriculum
	2. Population (physicians, nurses, medical students)
	3. Requirements (technical/didactical)
	4. Evaluation (yes /no)
	4.1 If yes, how?
	4.2 Results
	5. Advantages
	6. Disadvantages

### Patient and public involvement

This scoping review protocol and the final scoping review do not include patient recruitment or public involvement.

### Data analysis and presentation

Evidence will be presented in tabular form. A narrative summary will accompany the tabulated and/or charted results and will describe how the results relate to the reviews objective and questions. We will report findings in line with the “Preferred Reporting Items for Systematic Reviews and Meta-Analysis: extension for Scoping Reviews’ checklist [16].

## Discussion

The conduct of a scoping review enables to cover a broad range of published literature. The objective of this review is to display the diverse range of VR that has been applied in medical education yet. This review provides a transparent and reproducible search strategy in agreement with the updated JBI methodology for scoping reviews [14]. The methodology includes that literature will not be analyzed in terms of quality. The resulting review will comprise the identification of requirements for technical and didactical implementations, evaluation methods, and results. This will offer stakeholders to concisely detect crucial aspects for the design and development of VR applications and acquire knowledge about their integration into medical curricula. Findings will be disseminated through professional networks and submission of the resulting review to a peer-reviewed scientific journal.

## Supplementary Information

**Additional file 1.** PRISMA-P 2015 Checklist.

## Data Availability

Not applicable.

## Abbreviations

VR: Virtual reality
AI: Artificial intelligence

## References

[1] Bric JD, Lumbard DC, Frelich MJ, Gould JC. Current state of virtual reality simulation in robotic surgery training: a review. Surg Endosc. 2016;30(6):2169–78. Available from: https://link.springer.com/article/10.1007/s00464-015-4517-y. [cited 2021 Dec 15]. 10.1007/s00464-015-4517-y 26304107

[2] Izard SG, Juanes JA, García Peñalvo FJ, Estella JMG, Ledesma MJS, Ruisoto P. Virtual reality as an educational and training tool for medicine. J Med Syst. 2018;42(3):1–5. Available from: https://link.springer.com/article/10.1007/s10916-018-0900-2. [cited 2022 Jan 4]. 10.1007/s10916-018-0900-2 29392522

[3] Gutiérrez F, Tima, Pierce J, Vergara VM, Coulter R, Saland L, et al. The effect of degree of immersion upon learning performance in virtual reality simulations for medical education. 2007;155–60. Available from: https://ebooks.iospress.nl/publication/10735. [cited 2021 Dec 15]. 17377256

[4] Creutzfeldt J, Hedman L, Felländer-Tsai L. Cardiopulmonary resuscitation training by avatars: a qualitative study of medical students’ experiences using a multiplayer virtual world. JMIR Serious Games. 2016;4(2). Available from: /pmc/articles/PMC5203677/. [cited 2021 Dec 15]. 10.2196/games.6448 PMC5203677 27986645

[5] Real FJ, DeBlasio D, Beck AF, Ollberding NJ, Davis D, Cruse B, et al. A virtual reality curriculum for pediatric residents decreases rates of influenza vaccine refusal. Acad Pediatr. 2017;17(4):431–5. Available from: https://pubmed.ncbi.nlm.nih.gov/28126612/. [cited 2021 Dec 15]. 10.1016/j.acap.2017.01.010 28126612

[6] Burke SM. Cultivating critical thinking using virtual interactive case studies. J Pediatr Nurs. 2017;33:94–6. Available from: https://www.pediatricnursing.org/article/S0882-5963(16)30435-3/fulltext. [cited 2021 Dec 15]. 10.1016/j.pedn.2016.12.001 28139403

[7] Pottle J. Virtual reality and the transformation of medical education. Futur Healthc J. 2019;6(3):181. Available from: /pmc/articles/PMC6798020/. [cited 2021 Dec 15]. 10.7861/fhj.2019-0036 PMC6798020 31660522

[8] Birrenbach T, Zbinden J, Papagiannaki G, Exadaktylos AK, Müller M, Hautz WE, et al. Effectiveness and utility of virtual reality simulation as an educational tool for safe performance of COVID-19 diagnostics: prospective, randomized pilot trial. JMIR Serious Games. 2021;9(4):e29586. Available from: https://games.jmir.org/2021/4/e29586. [cited 2022 Jan 4]. 10.2196/29586 PMC8510143 34623315

[9] Walter S, Speidel R, Hann A, Leitner J, Jerg-Bretzke L, Kropp P, et al. Skepticism towards advancing vr technology – student acceptance of vr as a teaching and assessment tool in medicine. GMS J Med Educ. 2021;38(6). Available from: https://www.egms.de/static/en/journals/zma/2021-38/zma001496.shtml. [cited 2022 Jan 4]. 10.3205/zma001496 PMC8493843 34651058

[10] De Ponti R, Marazzato J, Maresca AM, Rovera F, Carcano G, Ferrario MM. Pre-graduation medical training including virtual reality during COVID-19 pandemic: a report on students’ perception. BMC Med Educ. 2020;20(1):1–7. Available from: https://bmcmededuc.biomedcentral.com/articles/10.1186/s12909-020-02245-8. [cited 2022 Jan 4]. 10.1186/s12909-020-02245-8 PMC7517753 32977781

[11] Haerling KA. Cost-utility analysis of virtual and mannequin-based simulation. Simul Healthc. 2018;13(1):33–40. Available from: https://journals.lww.com/simulationinhealthcare/Fulltext/2018/02000/Cost_Utility_Analysis_of_Virtual_and.6.aspx. [cited 2021 Dec 15]. 10.1097/SIH.0000000000000280 29373382

[12] Jerald J. The VR book: human-centered design for virtual reality. Preface, Chapter 2. Association for Computing Machinery and Morgan & Claypool; 2015.

[13] Mergen M, Junga A, Risse B, Valkov D, Graf N, Marschall B., Konsortium medical tr.AI.ning. Immersive training of clinical decision making with AI driven virtual patients – a new VR platform called medical tr.AI.ning. GMS J Med Educ. 2023;40(2):Doc18. 10.3205/zma001600. URN: urn:nbn:de:0183-zma0016002. Available from: https://www.egms.de/static/en/journals/zma/2023-40/zma001600.shtml. [cited 2023 May 31]. 10.3205/zma001600 PMC10285366 37361242

[14] Jiang H, Vimalesvaran S, Myint Kyaw B, Tudor Car L. Virtual reality in medical students’ education: a scoping review protocol. BMJ Open. 2021;11(5):e046986. Available from: https://bmjopen.bmj.com/content/11/5/e046986. [cited 2022 Jan 27]. 10.1136/bmjopen-2020-046986 PMC8160201 34039577

[15] Jiang H, Vimalesvaran S, Wang JK, Lim KB, Mogali SR, Car LT. Virtual reality in medical students’ education: scoping review. JMIR Med Educ 2022;8(1)e34860. Available from: https://mededu.jmir.org/2022/1/e34860. [cited 2022 Apr 8]. 10.2196/34860 PMC8851326 35107421

[16] Peters MDJ, Marnie C, Tricco AC, Pollock D, Munn Z, Alexander L, et al. Updated methodological guidance for the conduct of scoping reviews. JBI Evid Synth. 2020;18(10):2119–26. Available from: https://journals.lww.com/jbisrir/Fulltext/2020/10000/Updated_methodological_guidance_for_the_conduct_of.4.aspx. [cited 2022 Mar 15]. 10.11124/JBIES-20-00167 33038124

[17] Piper C. System for the unified management, assessment, and review of information (SUMARI). J Med Libr Assoc. 2019;107(4):634. Available from: /pmc/articles/PMC6774554/. [cited 2022 Mar 2].

[18] Tricco AC, Lillie E, Zarin W, O’Brien KK, Colquhoun H, Levac D, et al. PRISMA extension for scoping reviews (PRISMA-ScR): checklist and explanation. Ann Intern Med. 2018;169(7):467–73. Available from: https://www.acpjournals.org/doi/abs/10.7326/M18-0850. [cited 2022 Mar 2]. 10.7326/M18-0850 30178033

